# Supplementary material for: Cancer and Potential Prevention with Lifestyle among Career Firefighters: A Narrative Review
Source: Cancers (Basel). 2023 Apr 24;15(9):2442. doi: 10.3390/cancers15092442 (PMC10177420; doi:10.3390/cancers15092442)
Supplement: Supplementary file 1 [file cancers-15-02442-s001.zip › cancers-2301958-supplementary.pdf]

Supplementary Table S1. Reviews on cancer among firefighters.

Author /year	Title	PMID	Location	Studies (N)	Purpose	Results	Conclusion	Limitations	strengths
**Laroche 2021 [20]**	Cancer Incidence and Mortality among Firefighters: An Overview of Epidemiologic Systematic Reviews.	33802629	USA, Canada, Australia, UK, Iran, Europe	11 systematic reviews (104 original studies)	To assess the conclusion consistency across the available systematic reviews on the cancer risk in firefighters.	A significant increase in the incidence of rectal, prostate, bladder, and testicular cancers as well as mesothelioma and malignant melanoma in firefighters compared to the general population. The SRs also indicate that death rates from rectal cancer and non-Hodgkin's lymphoma are higher among firefighters.	Consistent SR results suggest that several types of cancer may be more frequent in firefighters than in the general population.	There is currently little evidence available regarding the risk of cancer in female firefighters.	It is the first overview of the SRs on the risk of cancer in firefighters.
**Casjens 2020 [30]**	Cancer risks of firefighters: a systematic review and meta-analysis of secular trends and region-specific differences.	32306177	North America, Europe, Korea, Australia, New Zealand	25 cohort studies	To evaluate the cancer risks among firefighters in the time course and from different geographical areas.	The general cancer risk of firefighters was similar to the general population, but mSMR decreased over time. We observed an increase of mSIR for melanoma of the skin and prostate cancer as well as a decrease of mSIR for stomach cancer with later employment onset. For those cancer sites, we did not observe a secular trend of mSMRs. Regional differences between relative cancer risks were particularly observed for bladder cancer.	Among other things, innovative firefighting techniques and better personal protective equipment have provided a safer and healthier working environment for firefighters over time leading to a reduction of overall cancer incidence and mortality ratios. Increased general preventive medical checkups and possible additional screenings for firefighters might have led to more findings of malignant melanoma of the skin and prostate cancer in the recent past.	Small populations of firefighters, therefore low statistical power to analyze especially rare cancer types; low number of studies for each cancer type; length of follow-up might contribute to biased findings; too short follow-up times might prevent to observe cancers associated with older age; publications and data from other regions are missing	
**Jalilian 2019 [19]**	Cancer incidence and mortality among firefighters.	33802629	11 countries, dominantly in the USA (41%)	11 systematic reviews	To systematically review the literature on the association of firefighting occupation and	Significantly elevated SIREs for cancer of the colon, rectum, prostate, testis, bladder, thyroid, pleura, and malignant melanoma; significant SMREs of 1.36 and	Considering the significantly elevated risk of some cancers in this occupational group, we suggest improving preventive measures and securing adequate	Exposure information was not well-defined in the underlying articles and due to the small number of studies in most	This is the first comprehensive meta-analysis on firefighters and cancer that stratifies the outcomes by

					cancer incidence and mortality, overall and for specific cancer sites.	1.42 for rectal cancer and non-Hodgkin's lymphoma, respectively.	and relevant medical attention for this group. Further studies with more accurate and in-depth exposure assessments are indicated.	of cancers we could not further stratify our meta-analysis by different types of exposure surrogates.	cancer incidence and cancer mortality.
**Soteriades 2019 [29]**	Cancer Incidence and Mortality in Firefighters: A State-of-the-Art Review and Meta-Analysis	31759344	USA, Europe, Canada, New Zealand, Australia	49	A systematic literature review and meta-analysis was conducted on the association between firefighting and cancer.	Associations between firefighting and cancers of bladder, brain & central nervous system, and colorectal cancers, non-Hodgkin's lymphoma, skin melanoma, prostate, and testicular cancer; less consistent results for kidney, Hodgkin's lymphoma, leukemia, lymphosarcoma and reticulosarcoma, multiple myeloma, and pancreatic cancer; not any statistically significant associations for all other cancers evaluated	Although our meta-analysis showed statistically significant increased risks of either cancer incidence or mortality of certain cancers in association with firefighting, a number of important limitations of the underlying studies exist, which, precluded our ability to arrive at definitive conclusions regarding causation.	Small size of studies; short follow-up periods; difficulties obtaining data on occupational exposure; usually long latency periods associated with the development of different cancers.	-
**Lidoriki 2019 [60]**	Firefighting-Associated Cancers: Can Healthier Body Weight and Eating be Potential Countermeasures	30946290	USA	25 original studies, 4 meta-analyses	To identify the organ sites most consistently associated with an increased risk of cancer among firefighters.	Benefits are observed with respect to esophageal, colorectal, and head and neck cancer, and possibly a small benefit for prostate cancer. The highest adherence score to a Mediterranean diet was associated with a 14% lower risk of overall cancer mortality, a 18% lower risk of colorectal, and a 51% lower risk of head and neck cancer. The greatest and most consistent impacts of the Mediterranean diet were observed for colorectal cancer.	A healthy body weight and healthier eating are likely to be effective countermeasures for considerably reducing the risk of various firefighting-associated cancers, especially cancers of the digestive tract.	-	-

**Sritharan 2017 [26]**	Prostate cancer in firefighting and police work: a systematic review and meta-analysis of epidemiologic studies.	29149887	USA, Canada, Australia, Europe, New Zealand, Korea	26 firefighters and 12 police studies	To evaluate potential associations between firefighting and police occupations, and prostate cancer incidence and mortality.	For firefighter studies:<br><br>prostate cancer:<br><br>incidence mRE=1.17 (95% CI = 1.08–1.28, I^2 = 72%)<br><br>mortality mRE=1.12 (95% CI = 0.92–1.36, I^2 = 50%)	Small excess risks of prostate cancer were observed from firefighter studies with moderate to substantial heterogeneity. There is a need for further studies to assess unique and shared exposures in firefighting and police work.	Considerable heterogeneity between studies	The first to assess prostate cancer risk in both firefighting and police work, replete with subgroup analyses and assessments of study quality, heterogeneity, and publication bias.

mRE=meta-risk estimates, **SIR**=standardized incidence ratio, **SMR**=standardized mortality ratio, **SR**=systematic review
